# Phage ΦPan70, a Putative Temperate Phage, Controls *Pseudomonas aeruginosa* in Planktonic, Biofilm and Burn Mouse Model Assays

**DOI:** 10.3390/v7082835

**Published:** 2015-08-12

**Authors:** Angela V. Holguín, Guillermo Rangel, Viviana Clavijo, Catalina Prada, Marcela Mantilla, María Catalina Gomez, Elizabeth Kutter, Corinda Taylor, Peter C. Fineran, Andrés Fernando González Barrios, Martha J. Vives

**Affiliations:** 1 Department of Biological Sciences, Universidad de los Andes, Carrera 1#18A-12, Bogotá 111711, Colombia; E-Mails: av.holguin103@uniandes.edu.co (A.V.H.); garp1@leicester.ac.uk (G.R.); vivi.clavijo@gmail.com (V.C.); cat-prad@uniandes.edu.co (C.P.); marcelamantilla08@gmail.com (M.M.); gomez_katika@hotmail.com (M.C.G.); 2 T4 Phage Lab, the Evergreen State College, Olympia, Washington, DC 98505, USA; E-Mail: KutterB@evergreen.edu; 3 Department of Microbiology and Immunology, University of Otago, PO Box 56, Dunedin 9054, New Zealand; E-Mails: corinda.taylor@otago.ac.nz (C.T.); peter.fineran@otago.ac.nz (P.C.F.); 4 Grupo de Diseño de Productos y Procesos (GDPP). Department of Chemical Engineering, Universidad de los Andes, Carrera 1E# 19A-40, Bogotá 111711 Colombia; E-Mail: andgonza@uniandes.edu.co

**Keywords:** phage therapy, *Pseudomonas aeruginosa*, multiple drug resistance, phage genomics

## Abstract

*Pseudomonas aeruginosa* is one of the Multi-Drug-Resistant organisms most frequently isolated worldwide and, because of a shortage of new antibiotics, bacteriophages are considered an alternative for its treatment. Previously, *P. aeruginosa* phages were isolated and best candidates were chosen based on their ability to form clear plaques and their host range. This work aimed to characterize one of those phages, ΦPan70, preliminarily identified as a good candidate for phage-therapy. We performed infection curves, biofilm removal assays, transmission-electron-microscopy, pulsed-field-gel-electrophoresis, and studied the *in vivo* ΦPan70 biological activity in the burned mouse model. ΦPan70 was classified as a member of the *Myoviridae* family and, in both planktonic cells and biofilms, was responsible for a significant reduction in the bacterial population. The burned mouse model showed an animal survival between 80% and 100%, significantly different from the control animals (0%). However, analysis of the ΦPan70 genome revealed that it was 64% identical to F10, a temperate *P. aeruginosa* phage*.* Gene annotation indicated ΦPan70 as a new, but possible temperate phage, therefore not ideal for phage-therapy. Based on this, we recommend genome sequence analysis as an early step to select candidate phages for potential application in phage-therapy, before entering into a more intensive characterization.

## 1. Introduction

The World Health Organization (WHO) considers infections caused by Multiple Drug Resistant bacteria (MDR) a major public health problem [1]. One of the organisms contributing to this problem is *Pseudomonas aeruginosa* MDR, which is an opportunistic gram-negative pathogen. *P. aeruginosa* has the ability to colonize catheters, breathing tubes, and (infrequently) surgical tools; as a result, it can cause among others things, burn infections, bacteremia, septicemia, respiratory infections and rarely, recurrent endocarditis, mainly in immunosuppressed patients [2,3]. Furthermore, *P. aeruginosa* has a high prevalence in Intensive Care Units (ICUs) because of the ability to form biofilms on catheters, heart valves and breathing tubes [4]. In Bogotá, Colombia, *P. aeruginosa* is among the five most common organisms isolated in hospitals, according to data reported by the Group for the Control of Bacterial Resistance in Bogota (GREBO) [5] and, like elsewhere in the world, is resistant to multiple antibiotics.

In recent years, both the frequency of MDR microorganisms, as well as the number of antibiotics to which bacteria are resistant, has increased rapidly. A number of ICUs have reported incidents of *P. aeruginosa* infections resistant to every available antibiotic, leaving a patient with no viable treatments. The problem is compounded when the search for new antibiotics is expensive, arduous and not incentivized. It is often not profitable for pharmaceutical companies to develop new antimicrobials, especially when resistant strains are found within less than two years after their introduction [6].

After Frederick Twort and Felix d'Herelle discovered bacteriophages (phages; viruses that infect bacteria) in the 1910s, phages have been studied extensively for their potential to control bacterial infections [7,8]. However, due to the early lack of understanding about phages, various dubious claims about their efficacy were raised. In addition, the emergence of penicillin prompted research efforts, especially in the West, to focus on the identification and synthesis of antibiotics.

The difficulty of establishing the use of viruses as antimicrobial treatments, and the lack of clarity in defining the precedent for a regulatory process, are some of the factors that have impeded the development and extensive use of phages as therapeutics. A few countries have continued to use phages and have shown their efficacy in patients with infections caused by *P. aeruginosa* and *Staphylococcus aureus* MDR [9,10]. However, the regulatory bodies of the Eastern European countries that utilize phages do not have the same standards for approval of therapeutics that exist in other western countries [11]. Acceptance of phage products would also require phage production facilities and procedures to comply with the Good Manufacturing Practices (cGMP).

Responding to the calls of local physicians in desperate need of new alternatives for the control of MDR pathogens, several bacteriophages against *P. aeruginosa* MDR were isolated [12] and characterized to select the best candidates for phage therapy. Host ranges, genome size, morphology, its activity against biofilms and planktonic cells *in vitro*, and *in vivo* using mouse models and, lastly, genomic sequencing and annotation were performed for phage ΦPan70. Regular phage characterization involves all the aspects mentioned, and genomics is typically one of the later steps [13]. Genome sequencing and annotation is important for the assessing of presence of potential toxins and genes associated with the lysogenic cycle, which is one concern when the therapeutic phage application is discussed [13]. In this study, the genome analysis contradicted the previous collected evidence that suggested ΦPan70 as a good candidate for phage therapy, showing that researchers should consider genomics as an early step in the characterization of phages intended for therapeutic application.

## 2. Materials and Methods

### 2.1. Bacterial Strains

Three non-mucoid, MDR strains of *P. aeruginosa* from Hospital Federico Lleras de Ibagué were used in this study: *P. aeruginosa* P1, *P. aeruginosa* P3, and *P. aeruginosa* P4. *P. aeruginosa* P1 was resistant to ciprofloxacin, gentamicin, imipenem, meropenem, ceftazidime and had intermediate resistance to aztreonam and cefepime sensitivity; *P. aeruginosa* P3 was resistant to all antibiotics listed above; *P. aeruginosa* P4 showed resistance to ceftazidime, ciprofloxacin, gentamicin, imipenem, meropenem, intermediate resistance to cefepime and sensitivity to aztreonam (Table S1). Bacterial identification was carried out at the hospital and confirmed by 16S ribosomal DNA (rDNA) gene sequencing [14]. Four additional *P. aeruginosa* clinical strains (M1C1, Clínica del Prado Santa Marta; M4C1, Clínica Palermo Bogotá; M5C1, Clínica Palermo Bogotá; M8C1, Clínica Palermo Bogotá); two *Pseudomonas* sp. environmental strains (M6A1, M19A1); one *Escherichia coli* strain; and one *Salmonella enterica* subsp. *enterica* serovar Enteritidis strain, were used to test the host range of the phages.

### 2.2. Bacterial Culture Conditions

*P. aeruginosa* was grown in Lysogeny Broth-LB (NaCl 1% *w*/*v*, tryptone 1% *w*/*v*, yeast extract 0.5% *w*/*v*) and salts minimal medium (SMM) enriched with glucose (per liter: 3.5 g KH_2_PO_4_, 1 g (NH_4_)_2_HPO_4_, 1.2 g MgSO_4_, 5 g glucose, 12 mL trace elements; composition of the trace element solution per liter: 60 mg ferric citrate, 8.4 mg EDTA III, 2.76 mg CoCl_2_.6H_2_O, 15 mg MnCl_2_.4H_2_O, 8.4 mg zinc acetate, 2.67 mg Na_2_MoO_4_.2H_2_O, 3.3 mg H_3_BO_3_, 1.5 mg CuCl_2_.2H_2_O) [14]. Salt Magnesium (SM) buffer (10 mM Tris Base pH 7.4, 10 mM MgSO_4_, 0.02% *w*/*v* gelatin, 100 mM NaCl) [15] was used to store viruses. Soft LB agarose was used (LB plus 2.5 g per liter agarose SM8) to perform overlay assays [8]. Culture media and components were obtained from Oxoid Ltd (Basingstoke, Hampshire, England) unless otherwise stated.

### 2.3. Bacteriophage Isolation and Propagation

Twelve bacteriophages were initially isolated by enriching 1–2 g of horse feces (collected at, and kindly provided by, the Escuela de Caballería I in Bogotá) on 1× LB agar plates with 100 µg·mL^−1^ each of penicillin and chloramphenicol and incubated overnight at 37 °C. A single phage plaque per plate was picked and mixed with 4 mL of molten 0.25% agarose diluted in 1× TAE (diluted from a 50× stock with a composition per liter: 242 g Tris-base (Sigma Aldrich Pte Ltd, Singapore), 57.1 mL 100% acetic acid, 100 mL EDTA (Sigma Aldrich, Saint Louis, MO, USA) [8], 100 µg·mL^−1^ of penicillin and chloramphenicol and 400 µL of an overnight *P. aeruginosa* P4 culture and then poured onto LB plates. Plates were again incubated at 37 °C overnight. Individual plaques were picked into 1 mL of SM buffer and shaken gently overnight. From this suspension, bacteriophages were plated again; single plaques were re-suspended as described, filtered through 0.22 µm membrane filters, and stored at 4 °C. Lysates were made by growing *P. aeruginosa* P4 in 100 mL of LB until an OD_600nm_ of 0.7, where 500 µL of the phage suspension were added and incubated for 24 h at 37 °C. A 20 min centrifugation at 2800× *g* was performed to separate cellular debris from viruses. Then, ultracentrifugation was done at 22,000× *g* for 60 min to allow the phages to concentrate into a pellet and the supernatant was decanted away [16]. The phage pellet was re-suspended in 1 mL of SM buffer and filtered using a 0.22 µm membrane filter. These lysates were used in the host range determination.

### 2.4. Host Range, Plaque Morphology and Propagation of ΦPan70

Host range analyses were performed using spot tests with the strains listed earlier (*P. aeruginosa* P1, P3, P4, M1C1, M4C1, M5C1, M8C1; *Pseudomonas* sp. M6A1, M19A1; one *E. coli* and one *Salmonella* Enteritidis strains). One hundred µL of bacteria in the exponential growth phase was added to 4 mL of LB agarose and then poured onto LB plates. Plates were dried for 1 h at room temperature to form the overlay. Five µL of the phage suspension were spotted onto the bacteria overlay and dried for 15 to 20 min and allowed to incubate for 24 h at 37 °C. The results were recorded as positive or negative if a clear lysis zone was present or not [8]. Based on the host range and the ability to form clear and uniform plaques, one phage (named ΦPan70) was chosen for further characterization and testing *in vitro* and *in vivo*. Plaque morphology and titration was determined through serial dilutions followed by an overlay assay: 100 µL of an overnight bacterial culture and 100 µL of the phage lysate (1 × 10^9^ PFU/mL) are mixed together in 3 mL of soft LB agarose, poured onto an LB plate and incubated at 37 °C for 24 h.

### 2.5. Genome Size Estimation

High-titer ΦPan70 suspensions were obtained on exponential cultures of *P. aeruginosa* P4 by inoculating 100 µL of an overnight culture into 250 mL Erlenmeyer flasks containing 100 mL of LB, incubating at 37 °C and shaken at 250 rpm until they reached an OD_600nm_ of 0.2. Phages were added to the flasks (MOI 0.1) and incubated at 37 °C with slow shaking. After 24 h the lysates were centrifuged at 3400× *g* for 20 min and the supernatants were then filtered with 0.22 µm pore size syringe filters. Afterwards, the filtered supernatants were concentrated by centrifugation at 179,000× *g* for 30 min at 4 °C to obtain pellets that were allowed to re-suspend in 0.5 mL of SM buffer at 4 °C overnight. The phage suspensions were pooled together and centrifuged. The phage titer was determined by the double agar overlay assay explained earlier [8].

The genome size of ΦPan70 was estimated by pulsed-field gel electrophoresis (PFGE). Phage particles from the high-titer suspensions were immobilized in 0.7% (*w*/*v*) PFGE-grade agarose plugs and had their capsids degraded by introducing the plugs in tubes containing 2 mL of lysis buffer (1% *w*/*v N*-lauroylsarcosine, 0.2% *w*/*v* SDS, 0.1 mg·mL^−1^ Proteinase K) followed by incubation at 55 °C overnight. Lysis buffer was removed and 2 mL of wash buffer (20 mM Tris-HCl, 50 mM EDTA, pH 8.0) were added to the phage plugs; after 1 h, the wash buffer was decanted and three further wash steps were done, 30 min between each washing to assure the complete removal of the lysis buffer. Electrophoresis was performed in a 1% (*w*/*v*) PFGE-grade agarose gel, with TBE 0.5× as the running buffer using the following electrophoresis conditions: 6 V·cm^−1^, initial switch time 1 s, final switch time 6 s, included angle 120°, 14 °C and a run time of 18 h. Gel staining was performed using a 200 mL solution of 1X Gelred (Biotium Inc. Hayward, CA, USA), in distilled water for 30 min and then imaged using a Bio-Rad UV Transilluminator 2000 (Hercules, CA, USA) [16].

### 2.6. Transmission Electron Microscopy (TEM)

Phage lysates for TEM were made by ultracentrifugation (109,760× *g*, 30 min, 4 °C) from 5 mL phage lysates. Phage pellets were resuspended in ultrapure water overnight and then dialyzed in ultrapure water for 4 d to remove any residual salt. Carbon-coated copper grids (SPI-Grids™) were glow discharged, and 10 μL of the phage sample were placed on the grid and left for 1 min. The grid was blotted dry and stained with 10 μL of 1% phosphotungstic acid (pH 6.8). When necessary, the samples on the grids were washed five times in ultrapure water prior to staining. Grids were blotted dry and air-dried before examination in a Philips CM100 Transmission Electron Microscope (Philips/FEI Corporation, Eindhoven, The Netherland) [17].

### 2.7. Activity of ΦPan70 against Planktonic Cells

Infection curves were performed to test the effect of ΦPan70 in planktonic cells of *P. aeruginosa* MDR. The three strains, *P. aeruginosa* P1, P3 and P4 were cultured separately overnight in LB directly from 80 °C stocks and 300 µL were transferred to 30 mL of SMM and incubated at 37 °C for 20 h at 200 rpm. Then, 3 mL of this culture were transferred to another 30 mL of SMM and incubated under the same conditions to reach a bacterial concentration of ~10^6^ CFU·mL^−1^ for *P. aeruginosa* P1, ~10^8^ CFU·mL^−1^ for *P. aeruginosa* P3, and ~10^7^ CFU·mL^−1^ *P. aeruginosa* P4; these concentrations were established in a previous work, where mathematical models for phage titer optimization using planktonic cells resulted in different bacterial concentrations according to the strain [18]. Upon reaching the desired concentration of bacteria, 6.5 × 10^7^ PFU·mL^−1^ of phage suspension was added to the cultures. Bacterial and phage counts were measured; the bacterial titer was determined by taking aliquots, making serial dilutions with isotonic salt solution and plating on LB agar; the phage titer was determined by collecting aliquots that were mixed thoroughly with chloroform and, after 20 min, serially diluted and plated by the method of double layer agar described by Sulakvelidze and Kutter [8]. Each treatment was performed in triplicate. Controls consisted of bacterial cultures grown simultaneously in the same conditions, but without adding the phages.

### 2.8. Activity of Bacteriophages on Biofilms

The efficacy of ΦPan70 was tested and measured on biofilms of the three *P. aeruginosa* strains by using a colorimetric assay to show the amount of the remaining biofilm [19]. Aliquots of 10^6^ CFU·mL^−1^ inoculum previously cultured in SMM at 37 °C and 200 rpm, were diluted 1:20 using SMM; 190 µL were placed in each well of a polystyrene 96-well plate. Ten microliters of phage suspension were added (MOI of 0.001) at 0 min, 24 and 48 h after the bacteria inoculation (treatments) and then incubated statically at 37 °C. After 24 h post phage treatment, the wells were washed three times with 200 µL of PBS (0.1 M, pH 7.4; 137 mM NaCl, 2.7 mM KCl, 10 mM Na_2_HPO_4_, 1.8 mM KH_2_PO_4_) [20] and dried for 15 min before stained with 0.1% crystal violet. Unbound crystal violet was removed and the wells were again gently washed three times using 200 µL of PBS and allowed to dry for 15 min. Any remaining crystal violet that was bound to the biofilms was solubilized using 200 µL of 95% ethanol. Absorbance readings (OD_560nm_) were taken in a Bio-Rad 680 Microplate Reader. Each treatment was performed in triplicate and the results were compared using the *t*-test, with a significance *p*-value of 0.05, to the respective positive control (without phage).

### 2.9. Activity of Bacteriophages in the Burned Mouse Model

Animal handling followed the ethical standards recommended by Colombian guidelines for the care of laboratory animals (law 84 of 1989 and resolution 8430 of 1993, which in turn are based on the standards of the National Institutes of Health Guide for Care and Use of Laboratory Animals of the United States) [21,22]. The animal protocols used in this work were evaluated and approved by the Animal Use and Ethic Committee of the University of Andes. Swiss, female mice weighing 20–25 g from the vivarium of the University of the Andes were used. All animals were housed for four weeks in metal boxes (26 × 18.5 × 18 cm) in pairs and kept under a light-dark cycle of 12:12 h. Following the protocol reported by Heo *et al.* [23], groups of mice were infected with *P. aeruginosa* P4, which were subsequently treated with the optimal dose of ΦPan70 (6.5 × 10^7^ PFU·mL^−1^). The protocol consisted of anesthetizing the animals with ether, removal of 2 to 3 cm in diameter of hair on their back, followed by the burning of the skin area with water at 90 °C for 10 s and fluid replacement therapy with 0.8 mL 0.8% NaCl administered subcutaneously (s.c.). Bacterial infection was induced by inoculating 0.1 mL of the bacterial culture at an OD_600_ of 0.7, corresponding to 2.7 × 10^8^ CFU·mL^−1^, diluted 1:100 in PBS. When appropriate, 0.1 mL of the phage preparation with a concentration of 6.5 × 10^7^ PFU·mL^−1^ was administered s.c.

The experimental set up consisted of seven animal groups, four phage treatment groups and three control groups with five mice per group. Phage treatment groups varied depending on the time the phage was administered s.c. after the bacterial inoculation: 0 min, 45 min, 24 h and 48 h. The three control groups were comprised of the positive control, inoculated only with the bacteria, and two negative controls, one inoculated only with the phage and the other with PBS. The animals were observed daily during 15 d, recording every day their health condition and the number of dead animals. The health condition of the mice was rated on a scale of 4 to 0; 4: Healthy; 3: Small discharges from the eyes; 2: Discharge from the eyes, goose bumps and little activity; 1: Significant weight loss and no activity at all; and 0: Dead.

From the results obtained in the experiments described above, we conducted another set of experiments with the administration of a second dose of the phage preparation, 0.1 mL in a concentration of 6.5 × 10^7^ PFU·mL^−1^ s.c., 48 h after the first one. In this experiment, the second phage dose was administered only to the groups that had been treated with phage at 0 min and 45 min after bacterial inoculation. The health condition of the animals was assessed as previously described. Additionally, the activity of one mouse randomly selected from each group of mice was video recorded for 1 h a day and quantified by measuring the daily average velocity (cm·min^−1^) for a total of 15 d, using an EthoVision XT program (Noldus, Wageningen, The Netherlands ). Mice were transferred from their housing to a separate box containing a black floor during their 1 h recording. All data collected were analyzed and plotted using Prism software (Version 6. GraphPad Software Inc., La Jolla, CA, USA).

### 2.10. Genome Sequencing, Assembly and Annotation

DNA was isolated from ΦPan70 by phenol-chloroform extraction from a high titer phage stock (10^11^ PFU·mL^−1^) [8]. A 3 kb mate-pair library was prepared and Ion Torrent sequencing was conducted. For the genome assembly, shotgun reads and mate-pair reads were used with a genomic coverage of 60× and 20× respectively, these were assembled using the software Newbler v. 2.6. (Roche Diagnostics Corporation, Indianapolis, IN, USA). Gap filling was done by standard PCR and capillary sequencing of the amplified products (primers used to close gaps are listed in Table S2). Transfer RNA (tRNA) genes were searched using the program tRNAscan-SE [24], open reading frames (ORFs) were detected with GeneMark.hmm [25] employing heuristic models and glimmer 3.02 [26], both using the g3-iterated script and an interpolated context model (ICM) built from ORFs encoded in phage sequences similar to ΦPan70’s genome (identified after carrying out BLASTn with default parameters). Prior to building the ICM the selected ORFs were processed with the program cd-hit-est [27], in order to filter them for keeping only those that share less than 90% sequence identity and avoid redundant coding sequences (CDSs). Gene annotation was accomplished by employing the RAST server [28], BLASTp and by conserved domain identification with InterproScan. Comparison to other phage and prophage sequences was performed with standard BLASTn and by progressive genome alignments with Mauve [29]. wgVISTA software (version 1; Genomics Division of Lawrence Berkeley National Laboratory and US Department of Energy Joint Genome Institute) [30] was used for whole genome alignment between F10 and ΦPan70, with a global alignment algorithm. The genome sequence of ΦPan70 is available under GenBank accession number KJ959591.

## 3. Results

### 3.1. ΦPan70 is a Member of the *Myoviridae* Family

**Figure 1:**
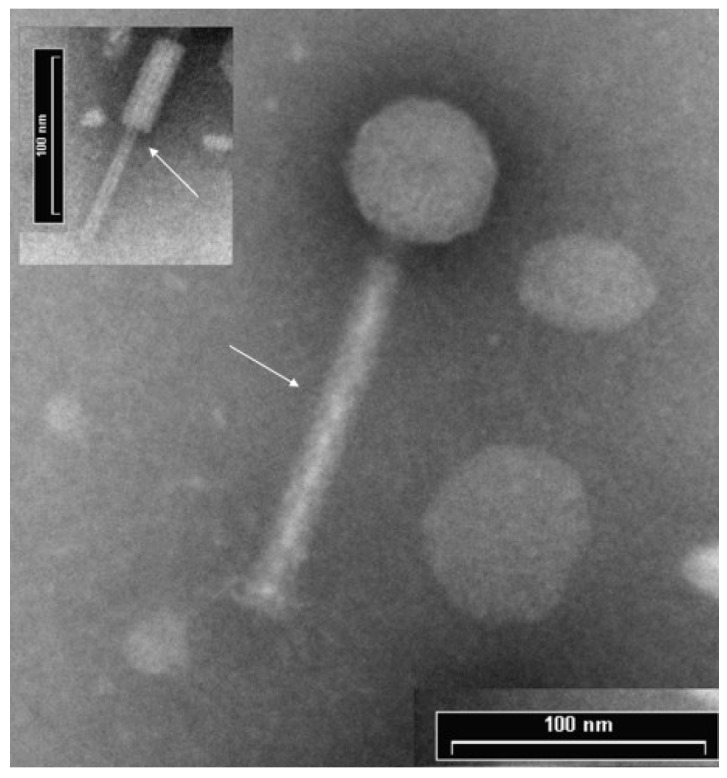
Transmission Electron Microscopy (TEM) of bacteriophage ΦPan70. TEM micrographs of negatively stained phage ΦPan70 showing an isometric head structure with a diameter of ~58 nm and a tail of 148 nm consisting of a neck, a contractile sheath and a central tube. The morphology of ΦPan70 corresponds to the *Myoviridae* family. Arrows show the extended tail sheath and, in the insert, the internal tube when the tail sheath is retracted.

Phage plaques were isolated and, based on the clear appearance, its ability to infect all three relevant *P. aeruginosa* MDR strains along with the other four clinical and two *Pseudomonas* environmental strains (M1C1, M4C1, M5C1, M8C1, M6A1, M19A1), and the lack of plaques in the *E. coli* and *Salmonella* strains, ΦPan70 was selected for further analyses. High-titer ΦPan70 phage stocks were prepared and the morphology determined using TEM. The micrographs showed a phage structure with a contractile tail and a capsid with icosahedral symmetry (Figure 1). The head width average was 58.91 nm, head height 58.18 nm, tail length 148.28 nm and total length of 206.46 nm. The genome size using PFGE was estimated as 35.9 kb and results from preliminary experiments showed that ΦPan70 genome was sensitive to restriction endonucleases like *Pst*I, indicating a double stranded DNA (dsDNA) genome. Therefore, ΦPan70 was assigned to the *Caudovirales* order and the *Myoviridae* family.

**Figure 2:**
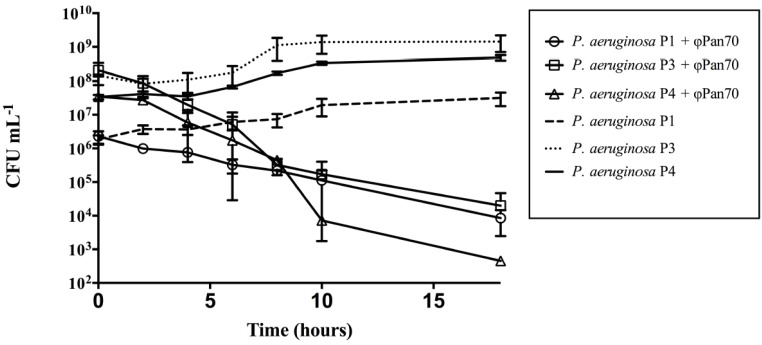
Bacteriophage ΦPan70 activity in planktonic cells of three different Multiple Drug Resistant bacteria (MDR) strains of *Pseudomonas aeruginosa*. Activity of ΦPan70 was assayed against planktonic cells of *P. aeruginosa* P1, P3 and P4. The corresponding control cultures for each strain (without phage) are also shown. Addition of the phage caused a reduction in the bacterial population, and the magnitude of this reduction depended on the particular strain. MOIs used varied depending on the strain (28, 1, and 2, respectively), according to a previous work on mathematical modeling for phage titer optimization [18]. Each treatment and control was performed in triplicate.

### 3.2. ΦPan70 Reduces Planktonic Cells

We wanted to determine how active would be ΦPan70 in killing planktonic *P. aeruginosa*. Figure 2 shows its activity against *P. aeruginosa* P1, P3 and P4 for up to 18 h after phage addition to cultures initially grown to a CFU/ml of 10^6^–10^8^. For *P. aeruginosa* P3 and P4 the viable count decreased by approximately 4–5 orders of magnitude, and *P. aeruginosa* P1 showed a 2–3 orders of magnitude reduction. Therefore, ΦPan70 demonstrated efficacy in antibacterial control against planktonic cells *in vitro*.

### 3.3. ΦPan70 Reduces Biofilm Formation and Existing Biofilms

**Figure 3:**
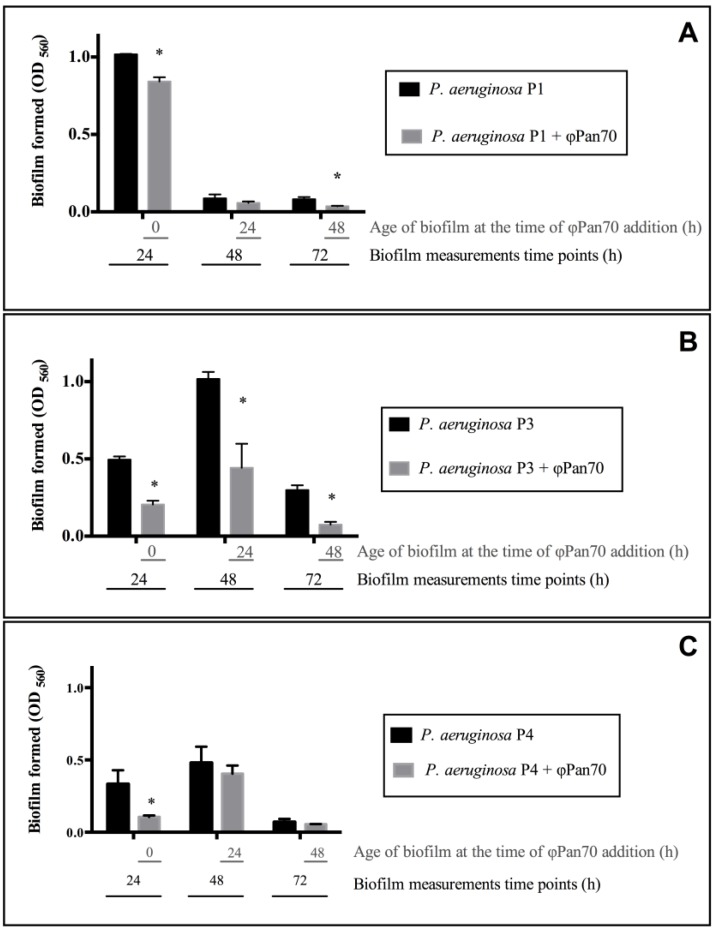
ΦPan70 prevents and removes biofilms of Pseudomonas aeruginosa MDR strains P1, P3 and P4. Bacterial biofilm formation with (**grey bars**) and without (**black bars**) ΦPan70. In all cases phage addition prevented or removed the biofilm of the three strains assayed, with significant differences (*p* < 0.05) compared to controls for some dataset (marked with asterisks) although different patterns were observed according to the strain. Phage was added at different times (0, 24 and 48 h) after bacteria inoculation in the 96-well plate. On the x axis, black numbers correspond to the time at which measurements of the biofilm formed were taken (24, 48 and 72 h), while the grey numbers correspond to the age of the culture or biofilm at which phage ΦPan70 was added. MOI was 0.001 in all cases. (**A**) *P. aeruginosa* P1; (**B**) *P. aeruginosa* P3; (**C**) *P. aeruginosa* P4. Each bar represents the mean ± SD of triplicate experiments.

Next, it was of interest to determine whether ΦPan70 was able to control *P. aeruginosa* growing in a biofilm state. Biofilms were assessed in a standard microtiter attachment assay. There were three different treatments: The first one, where ΦPan70 was added at 0 h (simultaneously with the bacterial inocula); the other two treatments received the phage at 24 and 48 h after bacterial inoculation to challenge the already formed biofilm. Biofilm density was assessed 24 h after the addition of phage in terms of absorbance (560 nm), and these biofilms were compared to controls that received no phage. Results are shown in Figure 3, and significant differences between the treatments and their respective control are marked with an asterisk. For *P. aeruginosa* P1, ΦPan70 induced a reduction in the biofilm of 17% at 0 h (*t*-test *p*-value: 0.003), 34% at 24 h (*t*-test *p*-value: 0.1338) and 55% at 48 h (*t*-test *p*-value: 0.0053). For *P. aeruginosa* P3, the phage reduced the biofilm in 59% at 0 h (*t*-test *p*-value: 9.8 × 10^−5^), 56% at 24 h (*t*-test *p*-value: 0.034) and 75% at 48 h (*t*-test *p*-value: 0.0004). In the case of *P. aeruginosa* P4, reduction in biofilm was 68% at 0 h (*t*-test *p*-value: 0.0153), 15% at 24 h (*t*-test *p*-value: 0.3584) and 21% at 48 h (*t*-test *p*-value: 0.2861). In summary, despite the differences among the three strains of *P. aeruginosa* in the biofilm cycle and the extent of the phage effect, ΦPan70 affects the biofilm by removing it or delaying its formation.

### 3.4. Phage-Therapy with ΦPan70 Improved Survival and Health in the Burned Mouse Model

**Figure 4:**
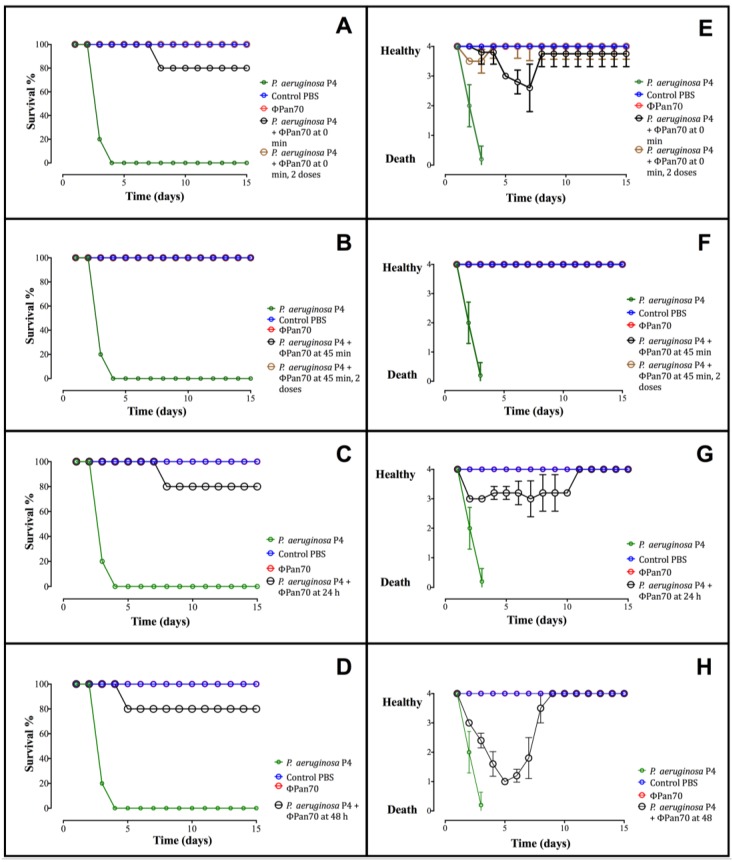
Survival percentage and health condition of mice treated with ΦPan70. All the phage treatments showed a dramatic reduction in the mortality of the mice and a general health condition improvement. Survival percentage was calculated according to the number of surviving animal recorded at each day of the experiment (Tables S3 and S4). The health condition was rated on a scale of 4 to 0; 4: Healthy; 3: Small discharges from the eyes; 2: Discharge from the eyes, goose bumps and little activity; 1: Significant weight loss and no activity at all; and 0: Dead animal. In an additional experiment, groups of mice treated at 0 min and 45 min received a second phage dose 48 h after the initial one. (**A**,**E**): Survival percentage and health condition, respectively, of the mice treated with ΦPan70 at the same time as bacterial inoculation (0 min). (**B**,**F**): Survival percentage and health condition, respectively, of the mice treated with ΦPan70 45 min after bacterial inoculation. (**C**,**G**): Survival percentage and health condition, respectively, of the mice treated with ΦPan70 24 h after bacterial inoculation. (**D**,**H**): Survival percentage and health condition, respectively, of the mice treated with ΦPan70 48 h after bacterial inoculation.

*P. aeruginosa* P4 was chosen for the animal infection model since ΦPan70 caused the greatest reduction in planktonic cells and was also somewhat effective in biofilm control. Bacterial challenge of the mice with *P. aeruginosa* P4 was performed on day 1 and the development of infection was followed for two weeks. Figure 4 shows the survival percentage as well as the scale of the general condition for the animals in each treatment. All positive controls without ΦPan70 treatment died between the third and fourth day. When ΦPan70 treatments were administered immediately after bacterial infection (0 min), four of the five mice survived (Figure 4A, Table S3). In the group treated 45 min after administration of the bacteria, all mice survived (Figure 4B). Similarly, in the groups that were treated at 24 and 48 h post infection, four of the five mice survived (Figure 4C,D). The health status of the mice was rated on a scale of 4 to 0 (4: Healthy; 0: Dead); the average of individual ratings is shown in Figure 4E–H. For the group of mice treated with the phage immediately after inoculation, the surviving animals showed a rating of 3 with the occurrence of eye secretions, which eventually disappeared (Figure 4E). The group of mice treated at 45 min after infection showed no symptoms of disease (Figure 4F). In the group treated at 24 h post infection, the mice were rated a 3 in the earlier days of follow up, whereas after the eighth day they resumed their normal condition and were classified as category 4 (Figure 4G). Similarly, individuals treated 48 h post-infection were initially classified as category 1 in the earliest days of follow up, but once they were treated with the phages they recovered and improved to a 4 (Figure 4H).

Another sign of infection was observed in the skin of the animals. The groups of mice treated at 0 and 45 min after infection showed severe lesion development on the back skin starting at day 8 and becoming severe during the 15 days of monitoring; this was observed externally and through histological sections. These individuals were unable to recover from the lesion within the observation period, although they survived. In individuals treated at 24 and 48 h post infection, the development of the skin lesion occurred (Figure S1A–C) but the lesions disappeared completely with the treatment of a single phage dose; their histological sections were indistinguishable from healthy epidermal tissue (Figure S1D).

We decided to assess the effect of applying a second dose of phages to the mice treated at 0 and 45 min after infection, to test the hypothesis that a second dose could prevent or help them to recover from the skin lesions. Tests were repeated for all treatment groups; the group of mice treated at 0 min was doubled and both subgroups received a single, initial phage dose; the second subgroup received a second dose 48 h after the initial one. The same approach was taken for the group of mice treated at 45 min after infection, whereas mice treated at 24 and 48 h post infection were given only a single dose. This second experiment replicated the data obtained previously, confirming the activity of phage ΦPan70 to control *P. aeruginosa* P4 in the burned mouse model: Positive controls died between the third and fourth day, and all individuals in each of the treatments and negative control groups survived (Figure 4A,B and Table S4). According to our hypothesis, groups treated at 0 and 45 min receiving the second phage dose fully recovered from the skin lesions. Regarding the health condition of the animals, scaling from 4 to 0 established before, all mice that received a second phage dose were optimal, given a rating of 4, within the 15 days of monitoring (Figure 4E,F). In order to have quantitative data on the general conditions of the animals in the second experiment, in addition to the qualitative ones, they were measured using the EthoVision XT program, which reflects the activity of the mice. A random individual mouse from each treatment and from the control group was filmed for 1 h each day of the experiment and the speed of their movements were calculated. The results were consistent with the data obtained in the qualitative scale from the first mice experiments showing that, in all cases after the phage treatment, mice recovered their normal activity (Figure S2). Additionally, no detrimental effect was observed in the animals inoculated only with the phage. In conclusion, treatment of mice with ΦPan70, even up to 48 h following *P. aeruginosa* infection, led to 80%–100% survival and improved the health condition of the mice.

### 3.5. Genome Sequencing, Assembly and Annotation

The genome assembly of phage ΦPan70 resulted in one 38.8 kb scaffold, which correlated closely to the preliminary PFGE estimation of 35.9 kb. This scaffold contained three contigs separated by a gap of 1190 bp between the first and the second contig, and a 115 bp gap between the second and third contig. From the alignment against database sequences we identified possible missing coding sequences (CDSs); appropriate primers were designed to PCR amplify them and the amplified products were sequenced. When these amplicons sequences were assembled, the ΦPan70 genome size was increased to 39,673bp. However, we reported it to the NCBI as a draft genome (accession number KJ959591) because: (a) there is an incomplete CDS at the right end, corresponding to a putative chitinase (CDS putative chitinase gb|KJ959591|:39092->39673 PAN70_gp062); and (b) there are two proteins that appear to have frame-shifts. In the first one (encoded by gene PAN70_*gp005*), we found that a single nucleotide is missing and it caused a division of the protein in two CDS. In the second protein (encoded by gene PAN70_*gp013*), there were two nucleotides missing in the sequence; one of the deletions is located within a low quality region, so this deletion could be a sequencing error. In both cases, the missing nucleotides belong to a homopolymer. It is known that the homopolymer resolution is one of the most common errors in the Ion Torrent and 454 sequencing technologies. Hence, these regions are under revision.

**Figure 5:**
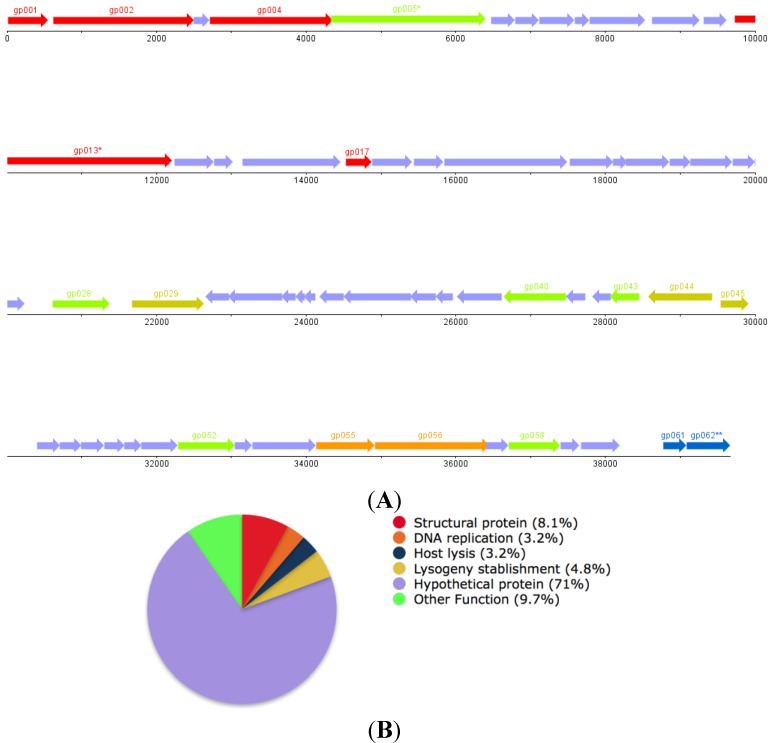
Genome map of *Pseudomonas aeruginosa* phage ΦPan70 and predicted gene functions. Bacteriophage ΦPan70 genome map (**A**); functions distribution pie chart (**B**); and protein functions predicted (**C**). In (**A**,**C**),* denotes disrupted ORF, potentially due to sequencing errors associated to the presence of homopolymeric segments. The pie chart in (**B**) shows the percentage of ORFs assigned to each functional group; “other functions” group contains predicted proteins unrelated to phage proteins. Coverage and *e*-values for predictions in (**C**) are shown in the Supplementary Material Table S5.

The phage draft genome has a GC content of 62%, similar to its *P. aeruginosa* host (66.6%). While no tRNA coding genes were found, 62 CDSs were identified, 47 of which are encoded on the positive strand (Figure 5A). The putative function of 18 of those CDSs was defined based on the similarity of the sequence of their protein products to proteins found in the NCBI protein database (for 17 CDSs), and the annotation of the CDS number 18, gp45, was supported by the identification of conserved domains characteristic of previously defined protein families (Figure 5B,C). Overall 71% of the protein products encoded in the genome of ΦPan70 were designated as hypothetical proteins, while the remaining had putative functions that were classified in the following groups: Virion structural proteins (8.1%), lysogeny-establishment proteins (4.8%), DNA replication proteins (3.2%), host lysis proteins (3.2%) and proteins with other function (9.7%). This last group comprises *gp005*, *gp028*, *gp040*, *gp043*, *gp052* and *gp058*, whose functions could not be classified into any of the functional modules typically found in phage genomes. No bacterial virulence proteins were found in the genome of ΦPan70. After scanning the protein products for the presence of conserved domains, it was found that *gp44* has a DNA binding domain and a peptidase S24/S26A/S26B/S26C domain (pfam *e*-values 2.8E–11 and 1.4E–4, respectively) that are characteristics of the C1-like repressors found in different types of temperate phages. Also, in the predicted amino acid sequence of *gp45*, a domain characteristic of the lambda phage Cro repressor was detected (pfam *e*-value 4.8E–8). This suggested that ΦPan70 is likely to be a temperate phage that might be able to integrate into the genome of particular host bacteria and form a prophage. After doing a progressive genome alignment of the genome sequence of ΦPan70 to that of the *P. aeruginosa* temperate phage F10, a nucleotide identity of 64% was found between the two sequences (Figure S3). In addition, the genome of ΦPan70 was significantly similar to the sequences of prophages found in different strains of *P. aeruginosa*, such as the LESB58 strain (Figure 5C). Within the LESB58 genome, Prophage 1 and 2 had DNA sequences that were 55% and 46% identical to ΦPan70, respectively, and both prophage regions had integrase genes. In a previous study [31], PCR assays indicated that the Prophage 1 region was defective for excision while Prophage 2 was capable of making viable phage particles under particular conditions. Therefore, ΦPan70 is similar to a number of previously sequence temperate *P. aeruginosa* phages and is possibly a temperate phage.

**Table:** 

	ORF	**Function**
	*gp001*	Small Subunit DNA Packaging Dimer (*Pseudomonas* Phage F10)
	*gp002*	Large Subunit DNA Packaging Dimer (*Pseudomonas* Phage F10)
	*gp004*	Portal Protein (*Pseudomonas* Phage F10)
	gp005 *	Putative ClpP Protease (*Pseudomonas* Phage F10)
	gp013 *	Putative Tail Length Tape Measure Protein (*P. aeruginosa* LESB58)
	*gp017*	Tail Fiber Protein (*P. aeruginosa* PA21_ST175)
	*gp028*	DNA Modification Protein (*Desulfovibrio vulgaris* str. 'Miyazaki F')
	*gp029*	Site Specific Recombinase (*P. aeruginosa* ATCC 700888)
	*gp043*	LuxR Family Transcriptional Regulator (*P. aeruginosa* LESB58)
	*gp044*	Transcriptional Regulator (*P. aeruginosa* ATCC 25324)
	*gp045*	Repressor (*Pseudomonas syringae*)
	*gp052*	Rha Family Phage Regulatory Protein (*P. aeruginosa* LESB58)
	*gp055*	DNA Replication protein, DnaC (*P. aeruginosa* LESB58)
	*gp056*	Putative DnaB-like Replicative Helicase (*P. aeruginosa* LESB58)
	*gp058*	Putative Metallophosphoesterase (*Pseudomonas* PhageF10)
	*gp061*	Holin (*P. aeruginosa* LESB58)
	*gp62*	Chitinase (*P. aeruginosa* LESB58)
(**C**)		

## 4. Discussion

In this study we presented a new *P. aeruginosa* phage that showed an efficient bacterial control activity in the different assays. We will discuss first the experimental results that led to the selection of ΦPan70, followed by the genome findings that suggested it is a temperate phage, therefore making it unsuitable for phage-therapy.

Several lines of experimental evidence drove us to consider ΦPan70 as a good candidate to control *P. aeruginosa* infections: Clear plaque morphology, infections curves producing a high titer progeny, broad host range, and the results from the activity assay of ΦPan70 against planktonic cells that showed a significant reduction in the CFU·mL^−1^ compared to the control without phage. Within the span of 18.3 h (1,100 min), a single starting phage dose of 6.5 × 10^7^ PFU·mL^−1^, and a bacterial concentration of ~10^8^ CFU·mL^−1^, we observed different reductions in the bacterial counts, depending on strain, between three to five logarithmic units. This result represents a significant reduction in the bacterial population; for comparison, an *in vitro* study developed with a last-generation antibiotic using *P. aeruginosa* PA01, required high concentrations (ranging from 1 to 2 mg·mL^−1^ in one dose) to achieve a similar reduction of 5 orders of magnitude; however, this antibiotic concentration resulted in cell toxicity [32]. The three *P. aeruginosa* strains responded differently to the addition of the phage, in terms of the factor of population reduction. There are previous reports supporting that the phage-bacteria interaction is highly dependent on both the particular strain and the phage. For instance, Kay *et al.* detected no significant reduction with two lytic phages against *P. aeruginosa* and *E. coli* [33]. Sillankorva *et al.* [34] showed an 85% of bacterial biomass reduction using a *P. aeruginosa* close relative, *P. fluorescens*, and its lytic bacteriophage ΦS1. Transformed to percentage, ΦPan70 produced a reduction between 99.9% to ~99.999% depending on the strain.

The response of the biofilms to the phage challenge also differed from one strain to another. Results showed a significant reduction for *P. aeruginosa* P1 when phage was added at 0 min and at 48 h; for *P. aeruginosa* P3, the reduction was significant at 0 min, 24 and 48 h; for *P. aeruginosa* P4 a significant reduction was observed when the phage was added at 0 min. It is important to note that the strains exhibited different biofilm kinetics; *P. aeruginosa* P1 has a rapid biofilm formation, producing higher biomass than *P. aeruginosa* P3 and *P. aeruginosa* P4 at 24 h, but also a rapid disaggregation phase evidenced by an important biomass reduction at 48 h and 72 h of biofilm age. *P. aeruginosa* P3 and *P. aeruginosa* P4 had more biomass than *P. aeruginosa* P1 at 48 h of biofilm age. Similarly, Phee *et al.* in their biofilms assays established that phage activity could vary according to the circumstances of the biofilm, including the amount of exopolysaccharide produced [35]. In summary, as well as with planktonic cells, the response of the biofilm to the phage was strain-dependent.

*In vitro* results were promising and justified testing ΦPan70 performance *in vivo*. *P. aeruginosa* P4 was chosen among the three strains to proceed with *in vivo* assays, based on the efficacy of ΦPan70 with this strain in the planktonic assays and the initial reduction in the biofilm assays. Although it is usually referred as the burned mouse model, this animal model develops into a bacteremia as a result of a wound infection. Results from the burned mouse model for the control group of mice administered only with phages, showed that phage ΦPan70 has no toxicity or side effects; all mice survived, their general health conditions over time were as good as the control animals inoculated with saline solution. Previous studies have shown the same results; a phage therapy study subjected immunocompromised mice to *Staphylococcus aureus* and no negative side effects were observed; instead, the animals did experience a beneficial immune-function replacement effect [36]. Another study using purified capsid proteins from T4, one of the most well studied phage, showed stimulation of immune responses in both mice (*in vivo*) and human blood (*in vitro*), such as reactive oxygen species formation, inflammatory mediators or significant cytokine production [37]. Any negative side effects of the *in vivo* phage therapy has been so far associated with insufficient purification protocols [11] as well as cell lysis, potentially releasing endotoxins and/or other superantigens that simulate immune responses [38].

Phage treatments in the burned mouse model, with no exceptions, showed outstanding outcomes. All the phage treatments resulted in a dramatic reduction in the mortality, ranging between 0 and 20%; instead, the mortality of the control mice infected with *P. aeruginosa* was 100%. Kumari *et al.*, using the same mouse model, described how bacteria spread from the burned wound to the blood becoming a systemic infection, and causing the death of the animal [39]. In addition, Siebenhaar *et al.* showed the importance of the mast cells and the tumor necrosis factor in the immune control of *P. aeruginosa* [40]*.* In a previous study, Golkar *et al.* showed the effectiveness of a lytic phage to control *P. aeruginosa* in mice model of wound infection [41]. In the present study the phage was also able to rescue all mice in the burned model but the skin lesion appeared only in the 0 and 45 min experimental groups after the bacterial challenge. The animals did not recover from the skin lesion despite other health indicators being positive. We speculated that phages were able to prevent the bacteria spreading into the blood but a high concentration of bacteria remained confined at the inoculation site, causing a strong immunological response and cutaneous mastocytosis. In the treatments at 24 and 48 h after bacterial challenge, the infection advanced systematically, so the phages possibly could kill bacteria in the blood.

The draft genome of ΦPan70 is a dsDNA, small genome of around 39,673 bp in size, with a GC content of 62%, similar to the *P. aeruginosa* host (66.6%). Overall, 71% of the protein products encoded in the genome of ΦPan70 were designated as hypothetical proteins. The progressive genome alignment of the genome sequence of ΦPan70 to that of the *P. aeruginosa* temperate phage F10 resulted in 64% identity between the two sequences, showing that both genomes have a similar arrangement with two blocks of coding sequences located in one strand, separated by another set of coding sequences encoded in the opposite strand (Figure S3). Among the two regions containing coding sequences on the same strand in ΦPan70, one displays synteny from *gp001* to *gp027* when compared to the corresponding block on phage F10, while the other block from *gp045* to *gp061* shows synteny only for a subset of genes that encompasses *gp055* to *gp060*. Among the genes found in syntenic segments there are the structural genes identified, those associated to DNA replication and the putative metallophosphoesterase. The genomic region which contains CDS encoded on the opposite strand is where most of the sequence divergence between the genomes of ΦPan70 and F10 is located, nonetheless there are two sets of three syntenic genes located in that segment, one of them includes *gp031* to *gp033* while the other is comprised of *gp036* to *gp038* (all encode for hypothetical proteins). None of the proteins encoded in ΦPan70 that can be associated to lysogeny establishment functions seem to have a homologous counterpart in phage F10, even though proteins with high sequence identity were found in genomes of different *P. aeruginosa* strains (Figure 5). The genome of ΦPan70 was also significantly similar to the sequences of prophages found in different strains of *P. aeruginosa*, such as the LESB58 strain. Taking all the genomic data in consideration, ΦPan70 results similar to a number of previously sequenced temperate *P. aeruginosa* phages and, based on that evidence, we regard it as a possible temperate phage.

Around 71% of the ΦPan70 genome codes for hypothetical proteins. This results agree with previously reported findings [42], where it was described that it was not possible to assign a function to nearly 82% of the CDSs in the genomes of *P. aeruginosa* phages isolated at the time. No known virulence proteins were found in the ΦPan70 genome, which suggests that using this phage therapeutically should not lead to the delivery of new virulence determinants into the target bacteria. However, three lysogeny-related proteins were found: A site-specific recombinase (*gp029*), a C1-like repressor (*gp044*) and a Cro-like repressor (*gp045*). In addition, the genome of ΦPan70 showed high sequence identity and similar genomic structure to the *P. aeruginosa* temperate phage F10, as well to the sequences of two prophages in *P. aeruginosa* LESB58 and similar prophages in *P. aeruginosa* PACS458 and PACS171b. Phage F10 had been an “orphan” phage and hasn’t been classified within any of the well-defined groups, which suggests that, together with ΦPan70, they could form a new *Pseudomonas* temperate phage group. An interesting fact is that F10 has been reported as a siphovirus [43], while ΦPan70 has morphological features characteristic of the myoviruses. Therefore, the relatedness in terms of genome sequence coupled with the unrelated virion morphology could be a consequence of a recombination event that resulted in the exchange of virion structural proteins, a phenomenon widely reported for phage genomes known as mosaicism [44]. The ΦPan70 region containing the lysogeny-related genes differs the most from the genome of F10. However, the proteins encoded by these genes (*gp029*, *gp043* and *gp044*) are highly related to proteins encoded in prophages from different *P. aeruginosa* strains (*P. aeruginosa* ATCC 700888 and *P. aeruginosa* LESB58). ΦPan70 genome sequence was iterated using PHACTS software (version 0.3; Department of Computer Science, San Diego State University) [45] in order to assess its lifestyle likelihood; the results predicted a temperate lifestyle with a 0.540022 probability (0.038966 standard deviation; 200 replicates) (Table S6); according to McNair *et al.* [46], prediction is 99% precise if the averaged probability score of the predicted lifestyle is two standard deviations away from the averaged probability score of the other lifestyle, which was the case for ΦPan70 genome analysis. Additionally, the absence of tRNA genes supports the hypothesis of a temperate life style for ΦPan70 [47].

To the best of our knowledge, there are no studies with temperate phages in the burned mice model to compare with the results obtained here. Chung *et al.* proved that the presence of a temperate phage inhibits the twitching motility of *P. aeruginosa* becoming an effective means to control *P. aeruginosa* infections [48]. It is possible that ΦPan70 inhibited an essential bacterial function and, by doing so, controlled the infection but the actual mechanism was not explored.

This work highlights the importance of phage genome sequencing for its use as an alternative therapy, prior to further experiments. ΦPan70, which seemed to be an ideal candidate for controlling *P. aeruginosa* MDR infections, appears to be a temperate phage according to its genome annotation. Temperate phages should be avoided as therapeutic agents due to a variety of disadvantageous traits that could compromise the safety and health of a patient. For example, their ability to induce lysogeny rather than be obligatorily lytic potentially enhances the success of their bacterial host, making them resistant to related phages. Furthermore, they have the potential to introduce genes to the bacterial host in the process of lysogenic conversion [11]. Inversely, Krylov *et al.* have proposed using *vir* mutants of temperate phages that are both incapable of entering lysogeny and are able to effectively kill host cells that have been lysogenized by related temperate phages [49]. Isolation or engineering of *vir* mutants adds an additional step to the development of a phage therapeutic that would not be necessary if obligatorily lytic phages are selected. Besides, it would create another drawback since engineered phages will be considered as genetically modified organisms (GMOs), making the appropriate selection of phages the best alternative for the development of phage therapy.

In summary, ΦPan70 is presented here as a bacteriophage effective to control *P. aeruginosa* infections in planktonic, biofilms and in an animal model, but having potential genes for lysogenization in its genome. Based in our experience, the genome analysis should be an early step in the selection of candidate phages for application in phage-therapy, after the host range and growth parameters determinations, and before more laborious tests such as biofilm, TEM or *in vivo* assays. Advances in sequencing technologies make sequencing readily available and more affordable, allowing the early selection of better candidates for phage therapy, saving time and resources. Also, to the best of our knowledge, this is the first report of a phage isolated from tropical regions in America; the genome analysis suggests ΦPan70 as a new phage, opening the possibilities for discovery of new genetic resources.

## Supplementary Files

Supplementary File 1
