# Supplementary material for: Phage ΦPan70, a Putative Temperate Phage, Controls Pseudomonas aeruginosa in Planktonic, Biofilm and Burn Mouse Model Assays
Source: Viruses. 2015 Aug 12;7(8):4602–23. doi: 10.3390/v7082835 (PMC4576196; doi:10.3390/v7082835)
Supplement: Supplementary File 1 [file viruses-07-02835-s001.zip › Fig. S3.pdf]

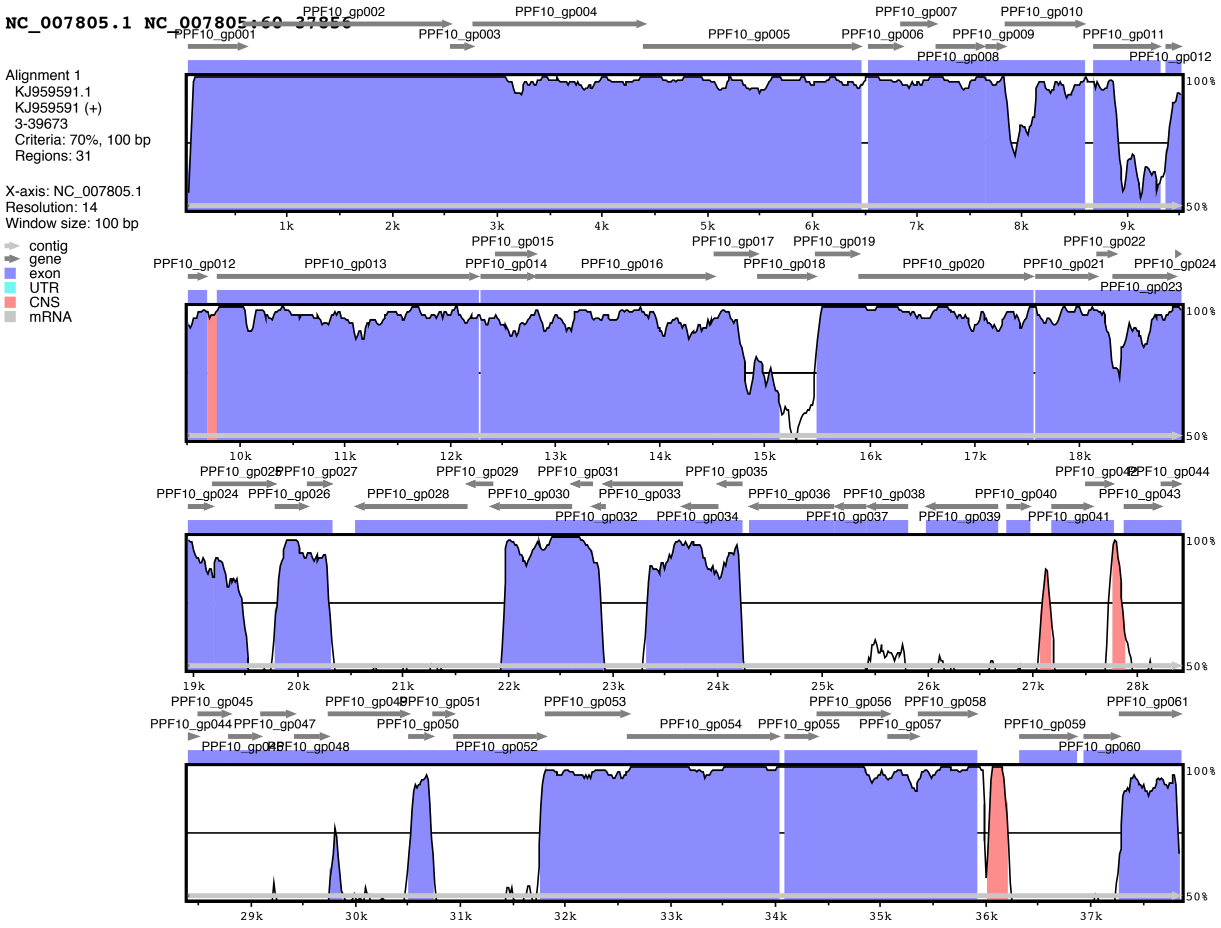

Figure S3. Whole genome alignment of F10 and ΦPan70. The alignment shows conserved regions between phage F10 and ΦPan70. The phage F10 (NC_007805.1) anotation is represented with arrows: light grey arrows represent contigs and dark grey arrows represent genes. The X-axis shows the base pares (Kbp). The Y-axis shows the conservation between the two genomes. The blocks in the alignment indicate the similarity of both phages in that region and the absence of those indicate they don’t share anything in that specific region.
