# Supplementary material for: Phage ΦPan70, a Putative Temperate Phage, Controls Pseudomonas aeruginosa in Planktonic, Biofilm and Burn Mouse Model Assays
Source: Viruses. 2015 Aug 12;7(8):4602–23. doi: 10.3390/v7082835 (PMC4576196; doi:10.3390/v7082835)
Supplement: Supplementary File 1 [file viruses-07-02835-s001.zip › Table S3.docx]

**Table S3.** Number of surviving mice over time, single phage dose experiments.

|  | **Number of surviving animals** | | | | | | | | | | | | | | |
| --- | --- | --- | --- | --- | --- | --- | --- | --- | --- | --- | --- | --- | --- | --- | --- |
| **Experimental group** | *Day 1* | *Day 2* | *Day 3* | *Day 4* | *Day 5* | *Day 6* | *Day 7* | *Day 8* | *Day 9* | *Day 10* | *Day 11* | *Day 12* | *Day 13* | *Day 14* | *Day 15* |
| Bacteria only (positive control) | 5 | 5 | 1 | 0 | 0 | 0 | 0 | 0 | 0 | 0 | 0 | 0 | 0 | 0 | 0 |
| Phage only (negative control) | 5 | 5 | 5 | 5 | 5 | 5 | 5 | 5 | 5 | 5 | 5 | 5 | 5 | 5 | 5 |
| Phage added at 0 minutes after bacteria inoculation | 5 | 5 | 5 | 5 | 5 | 5 | 5 | 4 | 4 | 4 | 4 | 4 | 4 | 4 | 4 |
| Phage added at 45 minutes after bacteria inoculation | 5 | 5 | 5 | 5 | 5 | 5 | 5 | 5 | 5 | 5 | 5 | 5 | 5 | 5 | 5 |
| Phage added at 24 hours after bacteria inoculation | 5 | 5 | 5 | 5 | 5 | 5 | 5 | 4 | 4 | 4 | 4 | 4 | 4 | 4 | 4 |
| Phage added at 48 hours after bacteria inoculation | 5 | 5 | 5 | 5 | 4 | 4 | 4 | 4 | 4 | 4 | 4 | 4 | 4 | 4 | 4 |
