# Supplementary material for: Phage ΦPan70, a Putative Temperate Phage, Controls Pseudomonas aeruginosa in Planktonic, Biofilm and Burn Mouse Model Assays
Source: Viruses. 2015 Aug 12;7(8):4602–23. doi: 10.3390/v7082835 (PMC4576196; doi:10.3390/v7082835)
Supplement: Supplementary File 1 [file viruses-07-02835-s001.zip › Table S6.docx]

Table S6. PHACTS analysis of ΦPan70 genome.

| FILE | LIFESTYLE | PROBABILITY | SD | LIFESTYLE | PROBABILITY | SD |
| --- | --- | --- | --- | --- | --- | --- |
| angela_phage.faa | Temperate | 0.557442557 | 0.024691475 | Lytic | 0.442557443 | 0.024691475 |
| angela_phage.faa | Temperate | 0.537262737 | 0.028372723 | Lytic | 0.462737263 | 0.028372723 |
| angela_phage.faa | Temperate | 0.525374625 | 0.03866663 | Lytic | 0.474625375 | 0.03866663 |
| angela_phage.faa | Temperate | 0.512687313 | 0.016668654 | Lytic | 0.487312687 | 0.016668654 |
| angela_phage.faa | Temperate | 0.531968032 | 0.037542742 | Lytic | 0.468031968 | 0.037542742 |
| angela_phage.faa | Temperate | 0.543056943 | 0.042917529 | Lytic | 0.456943057 | 0.042917529 |
| angela_phage.faa | Temperate | 0.548851149 | 0.033783372 | Lytic | 0.451148851 | 0.033783372 |
| angela_phage.faa | Temperate | 0.537262737 | 0.04871557 | Lytic | 0.462737263 | 0.04871557 |
| angela_phage.faa | Temperate | 0.536463536 | 0.05967864 | Lytic | 0.463536464 | 0.05967864 |
| angela_phage.faa | Temperate | 0.547452547 | 0.060169023 | Lytic | 0.452547453 | 0.060169023 |
| angela_phage.faa | Temperate | 0.536563437 | 0.033622976 | Lytic | 0.463436563 | 0.033622976 |
| angela_phage.faa | Temperate | 0.534265734 | 0.031326111 | Lytic | 0.465734266 | 0.031326111 |
| angela_phage.faa | Temperate | 0.565434565 | 0.041055038 | Lytic | 0.434565435 | 0.041055038 |
| angela_phage.faa | Temperate | 0.525574426 | 0.041387132 | Lytic | 0.474425574 | 0.041387132 |
| angela_phage.faa | Temperate | 0.527672328 | 0.04291908 | Lytic | 0.472327672 | 0.04291908 |
| angela_phage.faa | Temperate | 0.547252747 | 0.036043498 | Lytic | 0.452747253 | 0.036043498 |
| angela_phage.faa | Temperate | 0.54965035 | 0.032660526 | Lytic | 0.45034965 | 0.032660526 |
| angela_phage.faa | Temperate | 0.553946054 | 0.047829908 | Lytic | 0.446053946 | 0.047829908 |
| angela_phage.faa | Temperate | 0.544755245 | 0.039370301 | Lytic | 0.455244755 | 0.039370301 |
| angela_phage.faa | Temperate | 0.541558442 | 0.04191428 | Lytic | 0.458441558 | 0.04191428 |
|  |  | 0.540224775 | 0.03896676 |  | 0.459775225 | 0.03896676 |
