# Supplementary material for: Phage ΦPan70, a Putative Temperate Phage, Controls Pseudomonas aeruginosa in Planktonic, Biofilm and Burn Mouse Model Assays
Source: Viruses. 2015 Aug 12;7(8):4602–23. doi: 10.3390/v7082835 (PMC4576196; doi:10.3390/v7082835)
Supplement: Supplementary File 1 [file viruses-07-02835-s001.zip › Fig. S1.pdf]

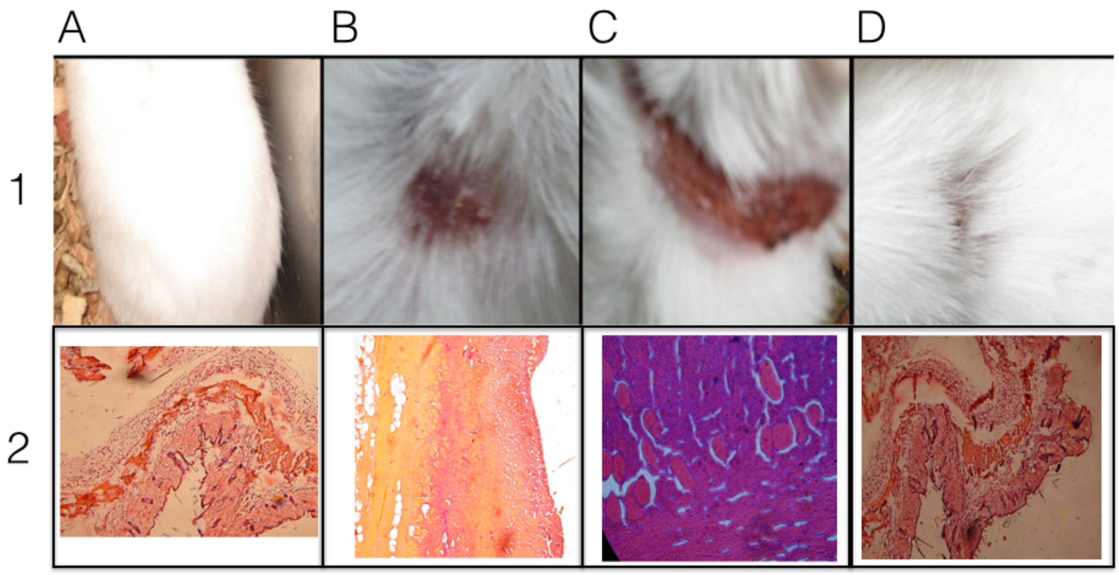

Fig. S1. ***Pseudomonas aeruginosa* lesion development and resolution in the mice treated with phage ΦPan70.**

Row 1: photographs of the lesion. Row 2: histology of the corresponding photograph in row 1. A) Skin before any treatment. B) Lesion developed at 24 hours after the bacterial challenge. C) Lesion at 48 hours after the bacterial challenge. D) Resolution of the lesion after phage treatment, photograph taken at day 15 after the bacterial challenge.
