# Supplementary material for: Phage ΦPan70, a Putative Temperate Phage, Controls Pseudomonas aeruginosa in Planktonic, Biofilm and Burn Mouse Model Assays
Source: Viruses. 2015 Aug 12;7(8):4602–23. doi: 10.3390/v7082835 (PMC4576196; doi:10.3390/v7082835)
Supplement: Supplementary File 1 [file viruses-07-02835-s001.zip › Fig. S2.pdf]

Fig. S2. Movement speed of mice treated with ϕPan70 at different times after *Pseudomonas aeruginosa* P4 infection

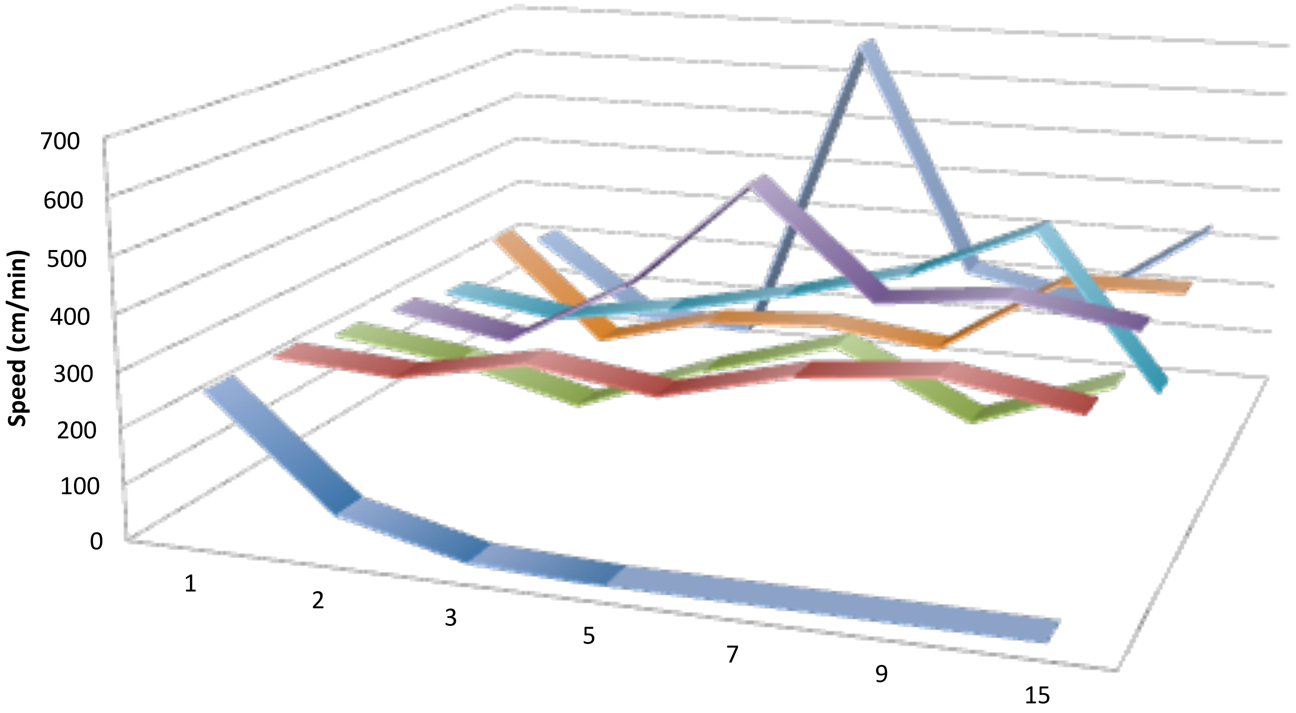

	1	2	3	5	7	9	15
bacteria only (positive control)	259,4	65	9,8	0	0	0	0
phage only	256,1	242	288,1	256,3	313,6	336	298,8
PBS (negative control)	234,5	205,3	147,5	235	302,6	190,4	278
0 min	225,1	185,4	312,6	525,5	324,1	350,7	315,2
45 min	201,8	170,3	208,6	259	320,3	424,3	132,9
24 h	262,1	63,5	124,7	138,2	111,7	265	268,5
48 h	211,4	54,8	45	679,8	215,9	169,3	333,8
